# Vaccination Patterns and Determinants of Influenza and Pneumococcal Vaccines Among COPD Patients in Shanghai, China: A Comparative Analysis of Differing Funding Strategies

**DOI:** 10.3390/vaccines13111119

**Published:** 2025-10-30

**Authors:** Xiaoqing Tang, Sichun Wang, Haifeng Xu, Haiying Tang, Fei Bian, Kuan Wan, Ruijie Gong, Wanjing Lin, Jingyi Ye, Qiangsong Wu, Qichao Zhang

**Affiliations:** 1 The Caohejing Community Health Service Centre in Xuhui District, Shanghai 200237, China; chjddyy@xh.sh.cn; 2 The Puxing Community Health Service Centre in Pudong District, Shanghai 200129, China; 18721575197@163.com; 3 Shanghai Fenxian Center for Disease Prevention and Control (Shanghai Fenxian Health Inspection Agency), Shanghai 201499, China; xuhaifeng@fengxian.gov.cn (H.X.); tanghaiying@fengxian.gov.cn (H.T.); bianfei@fengxian.gov.cn (F.B.); wankuan@fengxian.gov.cn (K.W.); 4 Shanghai Xuhui Center for Disease Prevention and Control (Shanghai Xuhui Health Inspection Agency), Shanghai 200237, China; cynthia-dt@sjtu.edu.cn (R.G.); jkzx_linwanjing@xh.sh.cn (W.L.); jkzx_yejingyi@xh.sh.cn (J.Y.)

**Keywords:** chronic obstructive pulmonary disease (COPD), influenza vaccine, pneumococcal vaccine, funding strategies, coverage, vaccination willingness, determinants

## Abstract

**Background:** Preventing and reducing acute exacerbations is a key objective in chronic obstructive pulmonary disease (COPD) management. Therefore, vaccination against influenza and pneumococcal disease is particularly important for this population. Under self-funded vaccination policies, the coverage rates for both vaccines among COPD patients in China are critically low. Since 2013, Shanghai has implemented a program providing one free dose of the 23-valent pneumococcal polysaccharide vaccine (PPV23) to residents aged 60 and above, whereas influenza vaccination remains self-funded. Few studies have compared influenza and pneumococcal vaccination coverage among COPD patients in China under these distinct funding strategies. **Methods:** This study used a stratified cluster sampling method to select COPD patients registered in the “Shanghai Community Chronic Disease Health Management System” from both urban (Xuhui) and suburban (Fengxian) districts of Shanghai. Data on demographic characteristics, medical history, physical examination results, behavioral risk factors, and vaccination records were extracted from the system. Vaccination records were verified using the “Shanghai Immunization Information System”. Descriptive analysis was conducted to assess influenza vaccine (self-funded, InfV) coverage during the 2023/2024 influenza season and cumulative PPV23 (government-funded) vaccination coverage among COPD patients. Logistic regression analysis was further employed to identify potential factors associated with InfV and PPV23 vaccination uptake in this population. **Results:** During the 2023/2024 influenza season, the influenza vaccination coverage under a self-funded policy was 5.87% among 1601 COPD patients in Shanghai, while the cumulative coverage of PPV23 under the government-funded program reached 52.15%. The willingness to receive PPV23 (60.40% vs. 27.55%; χ^2^ = 350.73, *p* < 0.001) and the uptake among willing individuals (86.35% vs. 21.32%; χ^2^ = 570.69, *p* < 0.001) were significantly higher under the free strategy compared to the self-funded InfV. For both vaccines, the primary reason for vaccine hesitancy was concern about adverse reactions, cited by over 50% of unwilling COPD patients. Multivariate analysis identified urban residence (aOR = 4.47, 95%CI: 2.86–6.98), prior PPV23 vaccination (aOR = 6.00, 95%CI: 3.43–10.49) and prior COVID-19 vaccination (aOR = 3.18, 95%CI: 1.79–5.66) as positive predictors of self-funded influenza vaccination. For PPV23 vaccination under the government-funded policy, significant factors included prior influenza vaccination (aOR = 6.89, 95%CI: 4.68–10.12), advanced age (aOR = 4.73, 95%CI: 3.68–6.09), and suburban residence (aOR = 0.37, 95%CI: 0.29–0.47). **Conclusions:** Influenza vaccination coverage among COPD patients in Shanghai remains critically low compared to the government-funded PPV23, highlighting the pivotal role of public funding. To address this disparity, urgent policy measures, including incorporating the influenza vaccine into publicly funded or health insurance reimbursement schemes, are essential.

## 1. Background

Chronic obstructive pulmonary disease (COPD) is a heterogeneous pulmonary condition characterized by persistent respiratory symptoms, including dyspnea, cough, and sputum production, as well as persistent airflow limitation [1]. The prevalence of COPD increases with age; globally, it affected 10.3% of adults aged 30–79 in 2019 [2]. In 2021, COPD was responsible for approximately 3.5 million deaths, accounting for about 5% of total global mortality, making it the third leading cause of death worldwide [3]. China, with its large and aging population, faces a particularly high burden, with a prevalence of 13.7% among adults aged ≥40 years old [4]. COPD prevention is thus an urgent public health priority. COPD patients experience 0.5 to 3.5 acute exacerbations per year. Acute exacerbations of COPD (AECOPD) represent the leading cause of death in this population [5]. Respiratory infections are the most common triggers of AECOPD; approximately 50% of cases involve upper respiratory viral infections, and 40–60% of patients have bacterial pathogens isolated from sputum [5]. Influenza virus and *Streptococcus pneumoniae* are frequently identified pathogens contributing to AECOPD, imposing a substantial disease burden on COPD patients [6].

A key objective in COPD management is to reduce and prevent acute exacerbations. Vaccination against influenza and pneumococcal disease is particularly important for achieving this goal. Administration of influenza vaccine (InfV) and pneumococcal vaccine (PV) has been shown to reduce the frequency of acute exacerbations [7], as well as associated hospitalizations and mortality among COPD patients [8,9,10,11]. Consequently, multiple international and national guidelines—including the Global Initiative for Chronic Obstructive Lung Disease (GOLD) [12], Healthy China Action (2019–2030) [13], and the Chinese Primary Care Guidelines for COPD Diagnosis and Management (2024) [14]—recommend routine InfV and PV vaccination for this population. Currently, developed countries with national immunization programs that include these vaccines demonstrate relatively high coverage rates among COPD patients [15]. For example, InfV coverage reaches 53.3% in France [16], 57.8% in Spain [17], and 66.7% in the United States [18]. Pneumococcal vaccine coverage in some European countries ranges between 10.8% and 32.5% [15]. In contrast, China implements a voluntary, self-funded vaccination policy for both InfV and PV, resulting in substantially lower uptake. Among patients hospitalized for AECOPD in China, coverage rates for both vaccines combined, InfV alone, and PV alone were reported to be 2.72%, 2.09%, and 1.25%, respectively [19].

The Chinese Primary Care Guidelines for COPD Diagnosis and Management (2024) [14] recommend that COPD patients receive one dose of influenza vaccine annually before each flu season and one dose of pneumococcal vaccine every five years. Multiple factors may influence vaccination uptake in this population, including patients’ awareness of diseases and vaccines, disease severity, vaccine availability, cost, affordability, geographical location, and the impact of the COVID-19 pandemic [19,20,21,22,23]. In September 2013, Shanghai launched a major public health program offering one free dose of 23-valent pneumococcal polysaccharide vaccine (PPV23) to all permanent residents aged ≥60 years old. By June 2020, an estimated 30% of older adults had received PPV23 under this policy. In contrast, influenza vaccination remains self-funded. This distinct difference in vaccination strategies—government-funded versus self-funded—provides a unique opportunity to examine the coverage and determinants of influenza and pneumococcal vaccination among COPD patients. Therefore, we selected COPD patients in Shanghai as a study population to observe vaccination coverage under different policy approaches and to further explore factors associated with InfV and PPV23 uptake. The findings aim to provide a scientific basis for optimizing vaccination strategies for COPD patients in China.

## 2. Methods

### 2.1. Study Design

Shanghai has prioritized the management of common chronic diseases, including hypertension, diabetes, and colorectal cancer, through initiatives aligned with the Shanghai Community Health Management Standards for Chronic Disease Prevention and Control (2017) [24]. In 2018, the city established the “Shanghai Community Chronic Disease Health Management System,” which achieved full coverage across all community health centers by 2021 [25,26]. Since 2023, this system has included a dedicated COPD management module to support community health centers in monitoring and managing COPD patients within their community.

**Figure 1:**
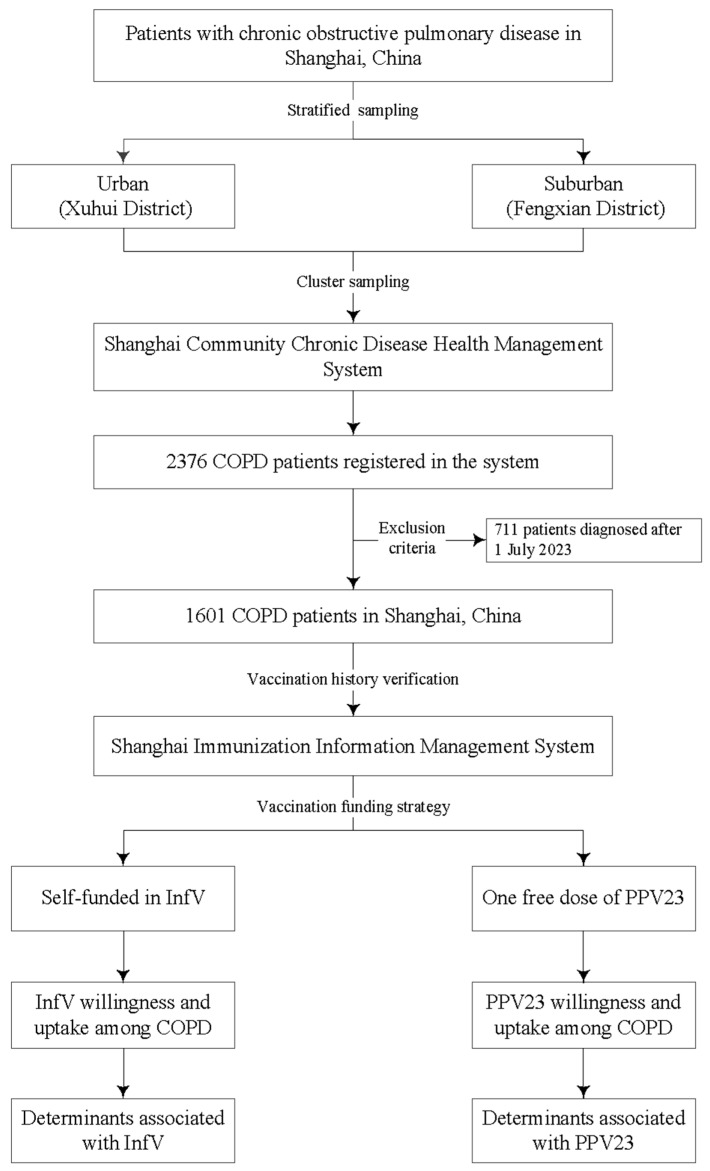
Flowchart of study design. Note: COPD, chronic obstructive pulmonary disease; InfV, influenza vaccine; PPV23, 23-valent pneumococcal polysaccharide vaccine.

This study employed a stratified cluster sampling method. First, stratification was performed based on the urban-suburban socioeconomic and healthcare gradient of Shanghai. Xuhui District was selected to represent a typical urban setting with more developed socioeconomic conditions and a dense healthcare network, while Fengxian District was selected to represent a suburban area with relatively lower socioeconomic indicators and a different healthcare resource profile. Within each stratum (district), a cluster sampling approach was implemented. The “clusters” were defined as all community health centers within the selected districts. All community health centers within these two districts were included in the study, thereby encompassing the entire registered COPD population from these centers. The study population consisted of COPD patients registered in the system at these centers. Systematically collected data were supplemented by verification of influenza and pneumococcal vaccination records through the “Shanghai Immunization Information System”. We accessed vaccination coverage for both vaccines during the 2023/2024 influenza season and identified factors associated with uptake under different funding strategies. The study design is summarized in Figure 1.

### 2.2. Study Individuals

The study population consisted of COPD patients managed by community health centers within the study sites. Inclusion criteria were (1) registration in the “Shanghai Community Chronic Disease Health Management System” of Xuhui or Fengxian District; (2) clinical diagnosis of COPD by a medical institution; and (3) completeness of key information, including a unique national identification number. Exclusion criteria included (1) non-registered COPD patients; (2) non-permanent residents of Shanghai; and (3) patients diagnosed with COPD after 1 July 2023.

### 2.3. Data Collection

COPD patient information was obtained from the “Shanghai Community Chronic Disease Health Management System,” including demographic characteristics, medical history, physical examination results, behavioral risk factors, vaccination records, and disease-related information. InfV and PPV23 vaccination records—both historical and those from the 2023/2024 influenza season—were verified using the “Shanghai Immunization Information Management System.”

### 2.4. Definitions

The InfV coverage was calculated as the percentage of COPD patients vaccinated during the 2023–2024 influenza season relative to the total number of patients diagnosed with COPD prior to the same season, expressed as follows.

InfV coverage = (Number of COPD patients vaccinated against influenza during the 2023–2024 season/Number of COPD patients diagnosed before the 2023–2024 season) × 100%.

The cumulative PPV23 vaccination coverage was defined as the proportion of COPD patients who had received at least one dose of PPV23 by the study cutoff date (1 July 2024) among all diagnosed COPD patients, calculated as follows.

Cumulative PPV23 coverage = (Number of COPD patients vaccinated with ≥1 dose of PPV23/Total number of diagnosed COPD patients) × 100%.

### 2.5. Statistical Analysis

Data were managed in Microsoft Excel (version 2019; Microsoft Corporation, Redmond, WA, USA) and analyzed with IBM SPSS Statistics (version 26.0; IBM Corporation, Armonk, NY, USA). Continuous variables were presented as mean ± standard deviation or median (IQR), while categorical variables were summarized as frequency (percentage). Group comparisons were performed using χ^2^ tests. Binary logistic regression was employed to identify factors independently associated with InfV or PPV23 uptake among COPD patients. Variables included in the logistic regression models were gender, age, residence, smoking status, drinking status, comorbid hypertension, comorbid diabetes, family history of COPD, COPD symptoms, and history of other vaccinations. Candidate variables were selected based on univariate analyses (*p* < 0.20) and clinical relevance. A backward likelihood ratio method was used for the multivariate model, retaining variables with *p* < 0.05. The overall model fit was significant (Omnibus test, *p* < 0.05), and the Hosmer–Lemeshow test indicated a good fit (*p* > 0.05). Results are presented as adjusted odds ratios (ORs) with their 95% confidence intervals (CIs). All tests were two-sided, and a *p* < 0.05 was considered statistically significant.

## 3. Results

### 3.1. Characteristics of Study Participants

We identified 2376 COPD patients registered in the “Shanghai Community Chronic Disease Health Management System” at community health centers in Xuhui and Fengxian Districts of Shanghai. After excluding 711 patients diagnosed after 1 July 2023, 1601 participants were ultimately included in the study. The study population was predominantly elderly, with 71.3% of participants being over 70 years old. The median age was 75 years (IQR: 70–80), and the median age at COPD diagnosis was 68 years (IQR: 62–75). Male patients accounted for 65.8% of the cohort. Geographically, nearly two-thirds of cases were from Fengxian District (64.46%). Comorbidities were highly prevalent: hypertension was present in 86.07% of patients, while diabetes was reported in 24.65%. Notably, the vast majority of patients were non-smokers (91.82%) and non-drinkers (86.95%). A positive family history of COPD was observed in 25.05% of cases. Regarding respiratory symptoms, 75.51% of patients reported none of the listed issues; among those with symptoms, chronic cough (12.51%) was the most commonly reported manifestation (Table 1).

### 3.2. InfV Vaccination Status Among COPD Patients During the 2023/2024 Influenza Season

**Figure 2:**
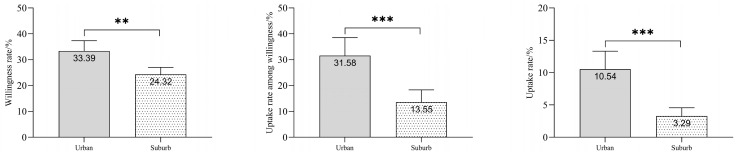
Comparison of Influenza vaccination willingness, uptake among willing individuals, and coverage between urban and suburban COPD patients in Shanghai, China. Note: COPD, chronic obstructive pulmonary disease; ** denotes *p* < 0.01; and *** denotes *p* < 0.001.

Under the self-funded influenza vaccination policy, among the 1601 participants, 441 expressed willingness to receive the InfV, yielding an intention rate of 27.55%. However, only 94 individuals actually received the InfV during the 2023/2024 season, yielding a vaccination coverage of 5.87%. These vaccinated individuals accounted for 21.32% of those who had expressed willingness. Only 4 out of the 94 COPD patients (4.26%) who received the InfV opted for simultaneous administration of the PPV23 vaccine. Regional disparities were observed: in the urban Xuhui District (*n* = 569), 190 patients indicated willingness (33.39%), and 60 were vaccinated (31.58% of willing individuals). In the suburban (*n* = 1032), 251 expressed willingness (24.32%), with 34 subsequently vaccinated (13.55% of willing individuals). Statistical comparisons showed significantly higher rates in urban COPDs for willingness (33.39% vs. 24.32%; χ^2^ = 8.43, *p* = 0.004), vaccination among willing individuals (31.58% vs. 13.55%; χ^2^ = 13.40, *p* < 0.001), and overall vaccination coverage (10.54% vs. 3.29%; χ^2^ = 30.45, *p* < 0.001), see Figure 2. None of the 1160 unwilling participants received InfV during the 2023/2024 influenza season. The primary reasons for non-vaccination were concern about adverse reactions (51.90%), perceived lack of necessity due to good health (15.09%), and inconvenience of access (2.50%). (Supplementary Table S1).

### 3.3. PPV23 Vaccination Status Among COPD Patients

**Figure 3:**
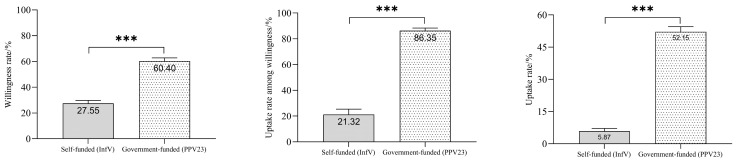
Comparison of influenza and pneumococcal vaccination willingness, among willing individuals, and coverage among COPD patients under differing funding strategies in Shanghai, China. Note: COPD, chronic obstructive pulmonary disease; InfV, influenza vaccine; PPV23, 23-valent pneumococcal polysaccharide vaccine; *** denotes *p* < 0.001.

**Figure 4:**
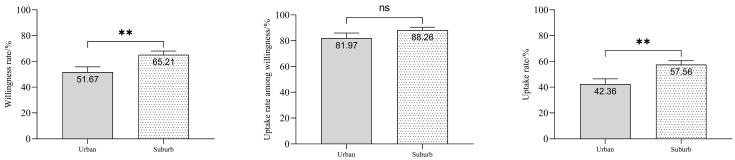
Comparison of PPV23 vaccination willingness, uptake among willing individuals, and coverage between urban and suburban COPD patients in Shanghai, China. Note: COPD, chronic obstructive pulmonary disease; “ns” indicates no significant difference (*p* > 0.05) between groups; ** denotes *p* < 0.01.

Under the government-funded program providing one free dose of PPV23 to older adults, among the 1601 participants, 967 expressed willingness to receive PPV23, yielding an intention rate of 60.40%. This rate was significantly higher than the intention rate observed for the self-funded influenza vaccine strategy (χ^2^ = 350.73, *p* < 0.001), see Figure 3. A total of 835 patients had received at least one dose of PPV23 by July 1, 2024, yielding a cumulative vaccination coverage of 52.15%. This vaccinated group accounted for 86.35% of those who had expressed willingness, significantly higher than the rate observed under the self-funded influenza vaccine strategy (χ^2^ = 570.69, *p* < 0.001), see Figure 3. Regional disparities were observed: in the urban Xuhui District (*n* = 569), 294 patients indicated willingness (51.67%), and 241 were vaccinated (81.97% of willing individuals). In the suburban Fengxian District (*n* = 1032), 673 expressed willingness (65.21%), with 594 subsequently vaccinated (88.26% of willing individuals). Statistical analysis revealed significantly lower rates in Xuhui District for both intention (51.67% vs. 65.21%; χ^2^ = 7.13, *p* = 0.008) and cumulative vaccination coverage (42.36% vs. 57.56%; χ^2^ = 11.03, *p* = 0.001), see Figure 4. The vaccination rate among willing individuals was also lower in Xuhui (81.97% vs. 88.26%), though this difference was not statistically significant (χ^2^ = 0.51, *p* = 0.475). None of the 634 unwilling participants had received PPV23. The primary reasons for non-vaccination were concern about adverse reactions (58.99%), perceived lack of necessity due to good health (13.56%), and inconvenience of access (3.15%). (Supplementary Table S2).

### 3.4. Determinants of InfV Administration Among COPD Patients Under Self-Funded Strategy

Univariate analysis revealed that residential area and prior PPV23 vaccination history were significantly associated with InfV uptake among COPD patients in Shanghai under the self-funded influenza vaccination policy. Patients residing in urban (Xuhui) areas demonstrated significantly higher odds of vaccination compared to those in suburban (Fengxian) areas (OR = 3.46, 95%CI: 2.24–5.34). A history of PPV23 vaccination was strongly associated with InfV administration (OR = 4.68, 95%CI: 2.71–8.09). No significant associations were found for other variables, including gender, age, comorbidities (hypertension or diabetes), smoking status, alcohol use, family history of COPD, COPD symptoms, or COVID-19 vaccination history. Multivariate analysis, adjusting for gender and age, identified urban residence (aOR = 6.27, 95%CI: 3.92–10.02), prior PPV23 vaccination (aOR = 5.99, 95%CI: 3.42–10.49), and a history of COVID-19 vaccination (aOR = 3.18, 95%CI: 1.79–5.66) as significant positive predictors of InfV vaccination. See Table 2.

### 3.5. Determinants of PPV23 Administration Among COPD Patients Under One Dose Free Strategy

Univariate analysis identified several factors significantly associated with PPV23 vaccination among COPD patients in Shanghai under the government-funded program providing one free dose of PPV23 to older adults. Prior influenza vaccination demonstrated the strongest association (OR = 4.15, 95%CI: 2.95–5.84). Patients aged over 70 showed substantially higher odds of vaccination (OR = 4.23, 95%CI: 3.34–5.35). Residence in Fengxian District (OR = 0.54, 95%CI: 0.44–0.67), presence of hypertension (OR = 1.44, 95%CI: 1.08–1.91), non-smoking status (OR = 0.62, 95%CI: 0.43–0.89), and absence of family history of COPD (OR = 0.79, 95%CI: 0.63–0.99) also showed significant associations. After adjustment for potential confounders (including gender, hypertension, smoking status, and family history), multivariate analysis identified prior influenza vaccination (aOR = 6.89, 95%CI:4.68–10.12), advanced age (aOR = 4.73, 95%CI: 3.68–6.09) and residence in suburban (aOR = 0.37, 95%CI: 0.29–0.47) as factors independently associated with PPV23 vaccination. See Table 3.

## 4. Discussion

Our study revealed that during the 2023/2024 influenza season, the InfV coverage under a self-funded policy among COPD patients in Shanghai was 5.87%, while the cumulative coverage of PPV23 under the government-funded program for older adults reached 52.15%. Compared with the 2017/2018 season under the same funding strategies, significant increases were observed for both InfV (5.87% vs. 0.4%) and cumulative PPV23 (52.15% vs. 22.8%) coverage among COPD patients [27]. The COVID-19 pandemic may have enhanced COPD patients’ acceptance of influenza and pneumococcal vaccines [28,29], as evidenced by the fact that 51.1% reported increased willingness of InfV due to the pandemic [23]. Our finding that a history of COVID-19 vaccination promoted InfV uptake further supports this assertion. During the 2023/2024 influenza season, InfV coverage among COPD patients in Shanghai (5.87%) exceeded the five-year average coverage (2015–2019) for the general population in Shanghai (1.4%) [30]. Similarly, the cumulative PPV23 vaccination rate among these patients (52.15%) was higher than the estimated coverage rate among older adults in Shanghai (30%) [24]. Similar patterns have been reported in other countries and regions. In Beijing, influenza vaccination coverage among COPD patients [23] was higher than that among influenza-like illness cases [31] (22.7% vs. 8.7%). In Spain, COPD patients showed higher influenza vaccine uptake than the general population (57.8% vs. 28.6%) [17]. In Korea, individuals with airflow limitation had higher vaccination rates than those without (61.2% vs. 41.8%) [32]. Three factors may contribute to higher vaccination coverage among COPD patients. First, patients often have greater health awareness and stronger motivation to prevent complications. Consistent with other studies showing higher PPV23 uptake among individuals with chronic conditions [33], our study found higher vaccination coverage among COPD patients with hypertension (53.41% vs. 44.39%). Second, recommendations from authoritative guidelines—such as those from ACIP, GOLD, and Chinese COPD management guidelines—have raised awareness among both patients and healthcare providers about the importance of vaccination [34]. Third, healthcare provider recommendation is one of the most influential factors in adult vaccination decisions [35]. Studies have shown higher influenza and pneumococcal vaccination coverage among COPD patients under the care of pulmonologists [36]. Recent efforts in China to promote adult vaccination prescriptions may further increase uptake through formal clinical recommendations [37]. However, influenza and PPV23 coverage among COPD patients in Shanghai—especially for InfV—remains lower. Health authorities should leverage integrated clinical and public health mechanisms to encourage vaccine prescribing by healthcare providers and improve vaccination coverage in this high-risk population [38].

In our study, PPV23 coverage under the government-funded program for older adults in Shanghai (52.15%) was significantly higher than influenza vaccine coverage under the self-funded policy (5.87%), a pattern consistent with findings from the 2017 survey under similar strategies (22.8% vs. 0.4%) [27]. In contrast, influenza vaccination coverage among COPD patients in Beijing exceeded PPV23 coverage (22.7% vs. 5.7%) [23]. This divergence reflects differences in funding strategies: Shanghai has provided free PPV23 to older adults since 2013 [24], while Beijing has offered free InfV to this population since 2007 [39]. Under the free strategy, PPV23 coverage in Shanghai was substantially higher than the national average for both the general population (30% [24] vs. 0.38% [40]) and COPD patients (52.15% vs. 0.8% [33]). We also observed higher cumulative PPV23 coverage among COPD patients aged 70 and above compared to younger patients, likely due to the age-restricted policy targeting individuals ≥60 years. However, even in Beijing, where InfV is freely offered, coverage among COPD patients (22.7% [23])—though higher than in Shanghai (5.87%)—remains below rates observed in developed countries (>50%) [16,17,18]. Our study found higher influenza vaccination coverage among urban COPD patients. This disparity may be attributed to greater financial capacity to self-fund vaccination in urban populations, whereas the vaccine cost likely posed an economic barrier for suburban patients. Furthermore, lower vaccination coverage in suburban areas could be exacerbated by poorer accessibility, including longer travel distances to clinics and insufficient public transportation [41], which compounds the associated time and financial burdens. The administration of both influenza and pneumococcal vaccines to COPD patients is recommended by major guidelines, including those from GOLD, ACIP, and NACI [34]. Reflecting this importance, several developed countries and regions have incorporated both vaccines into their National Immunization Program (NIP). According to WHO data, 58 countries and regions have included pneumococcal vaccines into their national immunization programs for high-risk groups [42]. With the availability of higher-valent PCVs, many high-income regions now recommend these vaccines or sequential schedules (PCV followed by PPV23) for high-risk populations [43]. Several key policies in China, including the Chinese Primary Care Guidelines for COPD Diagnosis and Management (2024) [14], the Chinese COPD Diagnosis and Treatment Guidelines [34], and the Healthy Shanghai Action—Chronic Respiratory Disease Prevention and Control Implementation Plan (2024–2030) [44], are expected to promote influenza and pneumococcal vaccination among COPD patients. However, a critical barrier remains: neither vaccine has been incorporated into China’s NIP, even for high-risk groups such as COPD patients. While national policy encourages local governments to provide free vaccination [45], implementation remains limited. For instance, during 2021–2022, only 256 counties reported such programs, covering just 1.43% of the national population [46]. In contrast, evidence from Shanghai demonstrates that government-funded strategies dramatically improve coverage. Therefore, given the substantial disease burden of COPD, we argue that there is an urgent need to establish fully funded or insurance-reimbursed vaccination programs for this population, pending their inclusion in the NIP.

In addition to higher vaccination coverage for PPV23 compared to InfV, we observed that the willingness to receive the government-funded PPV23 (60.40%) was significantly higher than for self-funded InfV (27.55%). Among those willing to vaccinate, the actual vaccination completion rate was also markedly higher for PPV23 (86.35%) than for InfV (21.32%). This disparity may be attributed to three primary factors. First, Shanghai’s sustained implementation and promotion of the free PPV23 policy for adults aged 60 and above since 2013 likely contributed to higher awareness [33] and uptake among the predominantly elderly COPD population (96.8% aged ≥60 years in our study). Consistent with other studies [19,33], our findings showed higher PPV23 coverage among older COPD patients. In contrast, no age-related advantage was observed for influenza vaccination under the self-funded policy, diverging from patterns seen in countries with free influenza vaccine programs [16,18]. Second, the free pneumococcal vaccine policy reduced financial barriers, enabling more COPD patients to be vaccinated. The requirement for annual influenza revaccination imposes a continuing financial burden on COPD patients, which can hinder uptake. Third, the successful implementation of Shanghai’s free PPV23 program for the elderly was greatly facilitated by high staff support, which promoted inter-agency collaboration and guaranteed vaccine supply, thereby enhancing accessibility for the target population [47]. Fourth, low disease awareness among COPD patients in China (5.7% [48]) may have limited their understanding of vaccine benefits—for example, perceiving influenza vaccines as offering limited protection—further suppressing willingness. The gap between high willingness and low actual uptake mirrors patterns observed with other vaccines, such as COVID-19 and HPV vaccines [49,50], and may be influenced by cost, accessibility, convenience, and knowledge. Consistent with other studies [33], a history of either influenza or pneumococcal vaccination was a significant promoting factor for uptake of the other vaccine among COPD patients in our cohort. This may be attributed to a better understanding of vaccine benefits and risks among these individuals, which positively influences their vaccination decisions. The top reasons for non-vaccination in our study were concern about adverse reactions and a perceived lack of need due to good health. However, with only 0.9% [48] of COPD patients in China aware of their diagnosis—despite being at high risk for influenza and pneumococcal disease—targeted health education is critical. Studies have shown that forgetfulness and a lack of knowledge about the vaccine’s effectiveness are significant barriers to influenza vaccination. Conversely, individuals with greater knowledge and more positive attitudes demonstrate a stronger intention to get vaccinated [51]. Evidence confirms the safety [52] and efficacy [11,53] of both vaccines in this population. Research indicates that intensive health education for COPD outpatients—including face-to-face recommendations, distributing health manuals, and sending SMS reminders—can significantly increase influenza vaccination rates (12.2% vs. 1.6%) [7]. Both hospital-based comprehensive interventions and community-based health education supported by information technology have been shown to improve vaccination coverage among COPD patients [54,55]. Furthermore, combined vaccination against influenza and pneumococcal disease is both synergistic [8] and effective in improving coverage [56]. However, our study found that the co-administration rate of these two vaccines among COPD patients was less than 5%. Given that the one-year readmission rate for COPD patients is as high as 42% [57], health authorities should collaborate with hospital-based COPD treatment departments and community-based follow-up management teams to implement integrated interventions. These should include disease and vaccine education, guidance on vaccination risks and benefits, the use of adult vaccine prescriptions [37], and vaccination reminder systems to improve overall vaccine uptake.

This study has several limitations. First, the analysis was based on data from COPD patients in Shanghai regarding influenza and PPV23 vaccination. Given the variations in vaccination policies and vaccine availability across different regions and countries, the generalizability of our findings to other settings may be limited. However, the primary objective of this study was to examine differences in vaccination coverage under distinct funding strategies. Second, although we observed higher cumulative PPV23 vaccination coverage compared to InfV under different funding strategies in Shanghai, the extent to which this disparity can be attributed to the free vaccination policy—as opposed to differences in vaccination schedules or patient perceptions of the two vaccines—requires further investigation. Third, the data collected for COPD patients were constrained by the information available in the “Shanghai Community Chronic Disease Health Management System”. Consequently, other potential factors influencing vaccination uptake, such as socioeconomic status, were not captured and should be explored in future studies. Fourth, although we intentionally selected Xuhui and Fengxian districts to represent urban and suburban settings in Shanghai to capture socioeconomic and healthcare gradients, the study population was restricted to COPD patients already enrolled in the official chronic disease management system within these two districts. These registered patients are likely to have stronger ties to primary care and be more health-conscious than non-registered individuals. This likely led to an overestimation of vaccination coverage and means our findings may not fully represent the entire COPD population in Shanghai or other regions.

## 5. Conclusions

COPD patients in Shanghai demonstrated substantially higher vaccination coverage under the government-funded PPV23 strategy (52.15%) compared to the self-funded influenza vaccine strategy (5.87%). This large difference shows that public funding plays a key role in improving vaccine coverage. To enhance immunization protection among COPD patients, we propose the following evidence-based recommendations. First, health authorities should consider incorporating the influenza vaccine into publicly funded or health insurance reimbursement schemes to address the financial barrier that currently limits uptake. Second, adopting a co-administration strategy for influenza and pneumococcal vaccines, coupled with the implementation of a healthcare provider-mediated reminder system, could improve accessibility and operational efficiency. The implementation of these targeted strategies is expected to narrow the vaccination gap, thereby strengthening the preventive role of immunization in the management of COPD.

## Figures and Tables

**Table 1:** Characteristics of study participants.

Population Characteristics	COPD Patients (*N* = 1601)	Proportion (%)/Value
Age Group (Years)		
Median (P25, P75)	1601	75 (70, 80)
≤70	460	28.73
>70	1141	71.27
Age at COPD diagnosis (years), Median (P25, P75)	1601	68 (62, 75)
Gender		
Male	1053	65.77
Female	548	34.23
Residence		
Urban	569	35.54
Suburban	1032	64.46
Comorbid diabetes		
No	1183	73.89
Yes	418	24.65
Comorbid hypertension		
No	223	13.93
Yes	1378	86.07
Smoking status		
No	1470	91.82
Yes	131	8.18
Drinking status		
No	1392	86.95
Yes	209	13.05
Family history of COPD		
No	1200	74.95
Yes	401	25.05
COPD symptoms *		
Dyspnea	27	1.58
Chronic cough	214	12.51
Sputum production	94	5.49
Wheezing and chest tightness	84	4.91
None of the above	1292	75.51

Note: COPD, chronic obstructive pulmonary disease. * Symptom data were collected as multiple-choice responses, resulting in 1711 answers from 1601 patients.

**Table 2:** Determinants of InfV administration among COPD patients in Shanghai, China.

Variable	Total	Number of Vaccinated Individuals	Univariate Analysis		Multivariate Analysis *	
			OR (95%CI)	*p*	aOR (95%CI)	*p*
Gender			0.79 (0.52–1.21)	0.281		
Male	1053	57				
Female	548	37				
Age			1.00 (0.63–1.59)	0.998		
≤70	460	27				
>70	1141	67				
Residence			3.46 (2.24–5.34)	<0.001	6.27 (3.92–10.02)	<0.001
Suburban	1032	34				
Urban	569	60				
Comorbid hypertension			1.11 (0.60–2.07)	0.737	/	/
No	223	12				
Yes	1378	82				
Comorbid diabetes			1.03 (0.64–1.65)	0.912	/	/
No	1183	69				
Yes	418	25				
Smoking status			1.20 (0.59–2.45)	0.612	/	/
No	1470	85				
Yes	131	9				
Drinking status			0.69 (0.34–1.40)	0.305	/	/
No	1392	85				
Yes	209	9				
Family history of COPD			1.29 (0.82–2.04)	0.275	/	/
No	1200	66				
Yes	401	28				
COPD symptoms			0.92 (0.54–1.58)	0.758	/	/
No	1292	77				
Yes	309	17				
Prior PPV23 vaccination			4.68 (2.71–8.09)	<0.001	5.99 (3.2–10.49)	<0.001
No	754	16				
Yes	847	78				
Prior COVID-19 vaccination			1.50 (0.88–2.57)	0.139	3.18 (1.79–5.66)	<0.001
No	392	17				
Yes	1209	77				

Note: COPD, chronic obstructive pulmonary disease; InfV, influenza vaccine; PPV23, 23-valent pneumococcal polysaccharide vaccine. The hyphen (“/”) denotes variables excluded from the multivariate model. * After adjusting for gender, age, the multivariate logistic regression model showed a good fit, as evidenced by a significant Omnibus Test (χ^2^ = 103.34, *p* < 0.001) and a non-significant Hosmer-Lemeshow Test (χ^2^ = 10.03, *p* = 0.123).

**Table 3:** Determinants of PPV23 administration among COPD patients in Shanghai, China.

Variable	Total	Number of Vaccinated Individuals	Univariate Analysis		Multivariate Analysis *	
			OR (95%CI)	*p*	aOR (95%CI)	*p*
Gender			1.06 (0.86–1.30)	0.612		
Male	1053	554				
Female	548	281				
Age			4.23 (3.34–5.35)	<0.001	4.73 (3.68–6.09)	<0.001
≤70	460	128				
>70	1141	707				
Residence			0.54 (0.44–0.67)	<0.001	0.37 (0.29–0.47)	<0.001
Suburban	1032	594				
Urban	569	241				
Comorbid hypertension			1.44 (1.08–1.91)	0.013		
No	223	99				
Yes	1378	736				
Comorbid diabetes			0.94 (0.75–1.17)	0.568	/	/
No	1183	622				
Yes	418	213				
Smoking status			0.62 (0.43–0.89)	<0.001		
No	1470	781				
Yes	131	54				
Drinking status			0.80 (0.60–1.07)	0.138	/	/
No	1392	656				
Yes	209	99				
Family history of COPD			0.78 (0.63–0.99)	0.036		
No	1200	644				
Yes	401	291				
COPD symptoms			1.05 (0.82–1.34)	0.719	/	/
No	1292	671				
Yes	309	164				
Prior InfV vaccination			4.15 (2.95–5.84)	<0.001	6.89 (4.68–10.12)	<0.001
No	1380	660				
Yes	221	175				

Note: COPD, chronic obstructive pulmonary disease; InfV, influenza vaccine; PPV23, 23-valent pneumococcal polysaccharide vaccine. The hyphen (“/”) denotes variables excluded from the multivariate model. * After adjusting for gender, hypertension, smoking status, and family history, the multivariate logistic regression model showed a good fit, as evidenced by a significant Omnibus Test (χ^2^ = 311.31, *p* < 0.001) and a non-significant Hosmer–Lemeshow Test (χ^2^ = 2.72, *p* = 0.606).

## Data Availability

The datasets used and/or analyzed during the current study are available from the corresponding author on reasonable request.

## Supplementary Materials

The following supporting information can be downloaded at: https://www.mdpi.com/article/10.3390/vaccines13111119/s1, Table S1. Reasons for Influenza Vaccine Hesitancy Among COPD Patients in Shanghai, China. Table S2. Reasons for 23-valent pneumococcal polysaccharide vaccine Hesitancy Among COPD Patients in Shanghai, China.
