# Supplementary material for: Vaccination Patterns and Determinants of Influenza and Pneumococcal Vaccines Among COPD Patients in Shanghai, China: A Comparative Analysis of Differing Funding Strategies
Source: Vaccines (Basel). 2025 Oct 30;13(11):1119. doi: 10.3390/vaccines13111119 (PMC12656854; doi:10.3390/vaccines13111119)
Supplement: Supplementary file 1 [file vaccines-13-01119-s001.zip › vaccines-3930658-supplementary.pdf]

Supplementary Materials

Table S1. Reasons for Influenza Vaccine Hesitancy Among COPD Patients in Shanghai, China.

Reasons for not willing to receive the InfV	Number of people (person-times)	Proportion（%）	Rank
Fear of adverse reactions	602	51.90	1
I'm in good health, so there's no need to get vaccinated	175	15.09	2
Inconvenient to get vaccinated	29	2.50	3
Poor vaccine efficacy	16	1.38	4
Contraindications to vaccination	9	0.78	5
Even if infected, it won't cause serious consequences	5	0.43	6
Others	324	27.93	-
Total	1,160	100.00	-

Table S2. Reasons for 23-valent pneumococcal polysaccharide vaccine Hesitancy Among COPD Patients in Shanghai, China.

Reasons for not willing to receive the PPV23	Number of people (person-times)	Proportion（%）	Rank
Fear of adverse reactions	374	58.99	1
I'm in good health, so there's no need to get vaccinated	86	13.56	2
Inconvenient to get vaccinated	20	3.15	3
Poor vaccine efficacy	11	1.74	4
Contraindications to vaccination	2	0.32	5
Even if infected, it won't cause serious consequences	1	0.16	6
Others	140	22.08	-
Total	634	100.00	-
